# Assessing Medical Tourism Services Quality Using SERVQUAL Model: A Patient’s Perspective

**Published:** 2018-01

**Authors:** Mohammad QOLIPOUR, Amin TORABIPOUR, Farzad FARAJI KHIAVI, Amal SAKI MALEHI

**Affiliations:** 1. Dept. of Health Services Management, School of Health, Ahvaz Jundishapur University of Medical Sciences, Ahvaz, Iran; 2. Dept. of Biostatistics and Epidemiology, School of Health, Ahvaz Jundishapur University of Medical Sciences, Ahvaz, Iran

**Keywords:** Medical tourism, Hospital, Service quality, Gap analysis

## Abstract

**Background:**

Continuous quality improvement of the hospital services is a basic requirement of medical tourism industry. The different dimensions of hospital services quality are assessed constantly to improve the service of medical tourism. The aim of this study was to determine the services quality of medical tourism in private and public hospitals.

**Methods:**

In this cross-sectional study, the quality of hospital services were assessed in view of 250 Iraqi tourists referred to Ahvaz private and public hospitals in 2015. Data were collected using a valid medical tourism SERVQUAL questionnaire (MTSQ). This questionnaire includes 8 main dimensions with 31 items. Finally, Mann-Whitney, Kruskal-Wallis and Wilcoxon tests were used to analyze the data.

**Results:**

The mean of age of patients was 39±2.2 yr. The mean of hospital length of stay was 3.87±1.36 days. The most patients were admitted to Orthopedics, Otorhinolaryngology, Obstetrics, and Gynecology departments, respectively. There was a negative gap in all of the dimensions of service quality in the studied hospitals (*P*>0.001). The highest and lowest quality gap was seen in the “exchange and travel facilities” (−2.63) and the “tangibles” (−0.68) dimension, respectively.

**Conclusion:**

There was a negative gap in all of the dimensions of service quality in the studied hospitals. Therefore, the hospital services quality is improved to attract the foreign patients.

## Introduction

Medical tourism is one expression of globalization (1). The term “medical tourism” is widely defined for the provision of medical care in collaboration with the tourism industry for patients who need medical services outside their country (1,2). For example, some of the Asian countries including India, Thailand, and Singapore are the major destinations for medical tourists (2, 3). The main reasons to choose these countries as a medical tourism destination are low cost medical services and experienced doctors (3, 4). In 2007, an estimated 50 000–120 000 patients of the United States travelled abroad to obtain medical care to Asia, South America, and Eastern Europe (5). In these countries, even uninsured patients based on their treatment can save about 15% to 85% of the costs (6).

According to the fourth economic and social development plan, Iran’s ministry of health must provide the necessary facilities for marketing of health services to earn more income (7). In Iran, opportunities such as high quality of medical services, low cost of medical services, access to new medical technology, and experienced health professionals can help to develop medical tourism industry(8).

Quality of medical services has a significant impact on attracting medical tourists (1, 2). Today the assessing and improving quality of services is one of the basic managerial tasks in the service sector (8). One of the most useful and applied methods to measure services quality is global SERVQUAL questionnaire. This common questionnaire used to measure the gap of services quality based on the patient’s expectation and perception (9). This tool was used to assess services quality. Most studies reported the service quality gap in private and public hospitals by SERVQUAL methods (10–13). In Iran, most studies reported a negative gap of hospital services quality based on the SERVQUAL method (8, 11, 12).

The aim of current study was to determine the services quality gap between medical tourism in Ahvaz public and private hospitals. Ahvaz is one of the metropolises of southern Iran bordered by Iraq. In This metropolis, thirteen public and private hospitals admit foreign patients as medical tourism hospitals (13).

## Materials and Methods

In this cross-sectional study, the quality of hospital services was assessed in view of 250 Iraqi tourists referred to Ahvaz private and public hospitals in 2015. Census data were collected using a valid medical tourism SERVQUAL questionnaire (MTSQ). This questionnaire was designed based on basic SERVQUAL model (14). The medical tourism SERVQUAL questionnaire included 31 items and 8 dimensions (tangibles, reliability, responsiveness, assurance, empathy, exchange and travel facilities, technical and infrastructure facilities and safety and security). A questionnaire was translated into Arabic and English. This tool can measure the gap between patients’ expectations and perceptions. The 5-point Likert scale was used to measure hospital services quality in view of patients. Likert points of perceptions and expectations were rated from 1 = strongly disagree to 5 = strongly agree.

Content validity of the questionnaire was confirmed using the Delphi technique and expert’s opinions (CVI=0.775). The construct validity of the questionnaire was confirmed using confirmatory factor analysis (RMSEA=0.032, CFI= 0.98, GFI=0.88). Reliability of the questionnaire using Cronbach’s alpha was confirmed (α=0.837 for expectation, α=0.919 for perception). The patient compleated the expectation questionnaires before admission and perceptions questionnaires after discharge, respectively. The gap of hospital service quality was measured using the following formula:

Gap=Perception−Expectation

If: E>P => Negative gap
If: E<P => Positive gap

Finally, the data were analyzed by SPSS (Ver.18 Chicago, IL, USA). Data were analyzed by non-parametric tests, including Mann-Whitney, Kruskal-Wallis and Wilcoxon tests. The significance level of study was 0.05.

This research was approved by Ethical Committee of Ahvaz Jundishapur University of Medical Sciences.

## Results

The mean of patients’ age was 39±2.2 yr. The mean of length of stay was 3.87±1.36 d. Orthopedics, Otorhinolaryngology, Obstetrics, and Gynecology departments had the most admitted patients respectively. About 82.8% of patients were uninsured. Out of 250 patients who participated in the study, 131 patients (52.4%) have not visited our hospitals before. There was no significant relationship between the gap in medical tourism service quality dimensions and patients’ demographic characteristics (Table 1).

**Table 1:** Demographic characteristics of the patients (n=250)

***Variable***		***Number***	***Percent***	***P-value***
Sex	Male	173	69.2	0.885
	Female	77	30.8	
Age (yr)	20 – 30	36	14.4	0.293
	30 – 40	41	16.4	
	40 – 50	85	34.0	
	50 – 60	88	35.2	
Language	Arabic	218	87.2	0.215
	Turkish	10	4.0	
	Kurdish	6	2.4	
	Farsi	7	2.8	
	Assyrian	9	3.6	
Having medical insurance	Yes	43	17.2	0.759
	No	207	82.8	
Education	University	102	40.8	0.712
	High school	83	33.2	
	Middle school or less	65	26.0	
Marital status	Single	70	28.0	0.613
	Married	180	72.0	
Admission based on hospital department	Orthopedics	43	17.2	0.993
	Otorhinolaryngology	41	16.4	
	Obstetrics and Gynecology	35	14.0	
	Urology	40	16.0	
	Ophthalmology	32	12.8	
	Cardiology	21	8.4	
	Neurology	17	6.8	
	Gastroenterology	12	4.8	
	General surgery	9	3.6	
There was a transportation problem	Yes	212	84.8	0.115
	No	38	15.2	
History of referring to the hospitals of Ahvaz	No	131	52.4	0.711
	Once	61	24.4	
	Twice	35	14.0	
	Three times and more	23	9.2	
Length of stay (days)	2	40	16.0	0.793
	3	76	30.4	
	4	57	22.8	
	5	40	16.0	
	6	28	11.2	
	7	9	3.6	

Assessing the gap between patients’ perception and expectation in eight dimensions of medical tourism service quality showed that the expectation of Iraqi medical tourists was more than their perception in all the dimensions; therefore, the quality gap of studied hospital services was negative.

**Fig. 1:**
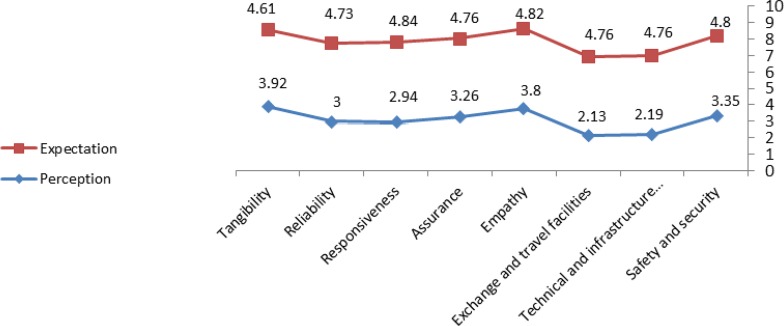
The gap between perception and expectation in eight dimensions

There was the highest and the lowest quality gap in the “exchange and travel facilities” dimension (−2.63) and the “tangibles” dimension (−0.68), respectively (Fig. 1).

All items had a negative gap. The highest quality gap was seen in “The website provides adequate information about treating illness” (G=−2.96) and “Payment facilities are easy and flexible” (G=−2.87) items, respectively. The lowest gap was seen in “neat and well-dressed personnel” (G=−0.66) and “modern and up-to-date equipment” (G=−0.68) items, respectively (Table 2).

**Table 2:** Mean and standard deviation of the scores of perception, expectation and quality gap for items of medical tourism service quality in Ahvaz hospitals

***Dimension***	***Item***	***Mean±S.D***		***Gap***
		**Perception**	**Expectation**	
**Tangibility**	Modern and up-to-date equipment	4.04±0.746	4.72±0.448	−0.68
	Visually appealing of physical facilities	3.80±0.759	4.52±0.501	−0.71
	Neat and well-dressed personnel	3.92±0.715	4.58±0.495	−0.66
**Reliability**	Providing services to patients with interesting	3.20±0.723	4.62±0.577	−1.42
	Accreditation of the hospital is globally accepted	2.73±0.780	4.86±0.384	−2.13
	Provide services at the time promised	2.96±0.851	4.78±0.418	−1.82
	Protecting patient records correctly by staff	3.10±0.886	4.65±0.562	−1.55
**Responsiveness**	Providing a detailed description of the provided services to patients by staff	2.85±0.790	4.77±0.501	−1.92
	Providing prompt service to patients by staff	3.12±0.834	4.91±0.290	−1.78
	Staffs’ continual willing to help patients	3.06±0.912	4.87±0.433	−1.81
	Patients’ transparent complaint process and responsiveness at the right time	2.73±0.753	4.80±0.404	−2.06
**Assurance**	Polite providers	3.24±0.689	4.76±0.425	−1.52
	The patient’s feeling of security in treating with providers	3.29±0.960	4.71±0.454	−1/42
	Providers have sufficient knowledge to answer the patient	3.08±0.765	4.88±0.330	−1.80
	Patient be ensured to the confidentiality of his/her information	3.42±0.871	4.68±0.582	−1.26
**Empathy**	Individual attention to patients	3.73±1.059	4.76±0.425	−1.03
	Understanding the specific needs of patients	3.68±0.949	4.78±0.418	−1.09
	Hospital staffs’ appropriate working hours for patients	3.97±0.937	4.92±0.272	−0.95
**Exchange and travel facilities**	Foreign exchange facilities are provided within the hospital	2.00±1.012	4.72±0.554	−2.72
	Payment facilities are easy and flexible	2.42±0.916	4.66±0.603	−2.24
	Adequate transportation facilities by the hospital	1.92±0.774	4.79±0.410	−2.87
	There are appropriate places near the hospital to stay	2.20±0.872	4.89±0.376	−2.69
	24h internet connectivity inside the hospital	2.53±0.953	4.49±0.782	−1.96
**Technical and infrastructure facilities**	The website provides adequate information on treating illness	1.87±0.852	4.82±0.382	−2.96
	Guaranteed reservation by the hospital	2.05±0.985	4.85±0.356	−2.80
	There is an office with administrative and commercial facilities for patients and their relatives	2.12±0.906	4.76±0.495	−2.64
	There are translation services in hospitals to facilitate personal relations and translate patients’ medical records	2.22±0.922	4.65±0.630	−2.43
	The doctors and nurses speak English / Arabic well	2.36±0.969	4.96±0.206	−2.59
	Providing safe medication services in a hospital	3.59±0.985	4.92±0.278	−1.32
**Safety and security**	Observe patient safety principles in the provision of technical services in a hospital (injections, dressing up, nursing services and medical examinations, etc.)	3.40±1.186	4.78±0.415	−1.38
	There is enough safety in the prevention of hospital events includes falling out of bed, stumble, etc.	3.06±1.022	4.71±0.454	−1.65

According to Table 3, the gap was negative and statistically significant, in all dimensions (*P*<0001). The highest gap was reported in “exchange and travel facilities” dimension (G=−2.63) and lowest gap was reported in “tangibility” dimension (G=−0.68) (Table 3).

**Table 3:** Mean and standard deviation of the scores of perception, expectation and quality gap for dimensions of medical tourism service quality in Ahvaz hospitals

***Dimension***	***Mean ± S.D***		***Gap***	***P-Value***
	**Perception**	**Expectation**		
Tangibility	3.92±0.696	4.61±0.412	−0.68	0.0001
Reliability	3.00±0.731	4.73±0.386	−1.73	0.0001
Responsiveness	2.94±0.737	4.84±0.346	−1.89	0.0001
Assurance	3.26±0.727	4.76±0.376	−1.50	0.0001
Empathy	3.80±0.917	4.82±0.327	−1.02	0.0001
Exchange and travel facilities	2.13±0.800	4.76±0.405	−2.63	0.0001
Technical and infrastructure facilities	2.19±0.800	4.76±0.401	−2.56	0.0001
Safety and security	3.35±0.978	4.80±0.334	−1.45	0.0001

## Discussion

The aim of this study was to determine the services quality of medical tourism in hospitals. According to SERVQUAL model, service quality gap was determined by measuring the differences between patient’s expectation and perception (10, 11). There was the negative gap for all dimensions of service quality in studied hospitals that providing medical tourism services. This means that medical tourists’ expectations were more than their perceptions about quality of medical service and they were not satisfied with hospital services.

In this study, tangibles (including physical evidence) had the lowest negative gap of services quality between other dimensions. In this dimension, there was a significant difference between perception and expectation of patients. The patients’ expectation was more than their perception in “tangibles” dimension about hospital services (12). In American medical centers, the gap between patients’ perception and expectation of service quality was also negative, but this gap was not statistically significant (15). Hospitals can play a key role in attracting medical tourists using advanced and standardized medical equipment and devices (16).

In the dimension of reliability, the service quality gap was also negative. A negative and significant difference between patients’ expectation and their perception in reliability of hospital services (10). Naqavi et al. reported a gap in service quality in Iranian hospitals (16). Reliability is one of the important dimensions of services quality. This dimension includes doing duties in accordance with the commitments, interesting employees in doing tasks and service provision, doing services correctly at the first visit of patients, providing the services on promised time, maintaining records of clients accurately, and increase trust between hospitals and medical tourists (17). Hospitals should improve the reliability of their services to increase patients’ loyalty.

In the current study, responsiveness dimension had also negative gap in view of patients. The mean of perception was less than expectation in responsiveness dimension in Iranian private hospitals (11). In American rehabilitation centers, the gap between the quality of services in responsiveness dimension was positive (18). Providing prompt service to patients is an important element in the responsiveness and patients had more satisfaction from hospitals that determine the time of treatment and provide prompt services (19, 20).

In assurance dimension, the difference between patients’ perception and expectations of hospital service was negative. The study in allergy clinics in Poland showed the negative gap in this dimension (21). In all developed countries, quality assurance committee of the hospitals assesses assurance of the services quality. Recently in Iran, hospitals establish quality improvement committees to control their clinical and services quality and initial steps have been taken to quality improvements (22).

In empathy dimension, the difference between patients’ perception and expectations of hospital service was negative. This difference was statistically significant. In Iran, a significant difference was indicated in empathy dimension of the hospital service quality (23). The gap between patients’ perception and expectation was positive in empathy dimension (24). Empathy is considered as the main domain of quality associated with non-clinical aspect of healthcare (25). To reduce the gap in empathy dimension, non-medical expectations should be developed by hospitals. These non-medical needs include dignity, confidentiality, autonomy, and communication (26). There was a negative gap in “exchange and travel facilities” dimension in view of foreign patients. The quality gap of this dimension was negative in the Indian public and private hospitals (27). Existing payment facilities in hospitals have a massive impact on the patient satisfaction and most patients consider this item before traveling to the destination country; therefore, it is necessary to increase exchange and travel services in the hospital (28).

The study showed that there was gap between patients’ perception and expectation in technical and infrastructure facilities dimension and this dimension was unsatisfactory for medical tourists. Hospital staff had no international experience and could not speak English and other foreign languages. To increase the foreign patients’ confidence, only reputation and scientific knowledge of doctors and professional staff are not enough and all aspects of professional staff should be improved. In the safety and security dimension of medical tourism service quality, there was a negative gap between perception and expectations of patients. Therefore, patients were not satisfied in term of hospital safety and security. Personnel behavior, social security and communication variables were respectively the most important factors to attract medical tourist (29). Appropriate prescription is one of the patients’ safety strategies in hospitals. Prescription and medication management were appropriate in Iranian private hospitals (27). Patient safety is the key indicators of hospital accreditation and quality improvement. Promoting patient’s safety led to improving hospital performance (30, 31).

This study had several limitations: First, the generalization of study to other hospitals is a problem. Second, all studied patients were from Iraq.

## Conclusion

There was a negative gap in all of the dimensions of services quality in the studied hospitals in Ahvaz. This means that studied hospitals could not met the patients’ expectation. The highest gap in the medical tourism service quality was observed in “exchange and travel facilities” dimension and the lowest gap was observed in the “tangibles” dimension. Therefore, studied hospitals should improve their quality of services based on gap score of services quality and improve their weakness. Finally, hospitals should promote national and international standards of medical tourism.

## Ethical considerations

Ethical issues (Including plagiarism, informed consent, misconduct, data fabrication and/or falsification, double publication and/or submission, redundancy, etc.) have been completely observed by the authors.
